# *LTRharvest*, an efficient and flexible software for *de novo *detection of LTR retrotransposons

**DOI:** 10.1186/1471-2105-9-18

**Published:** 2008-01-14

**Authors:** David Ellinghaus, Stefan Kurtz, Ute Willhoeft

**Affiliations:** 1 ZBH – Center for Bioinformatics, University of Hamburg, Bundesstrasse 43, 20146 Hamburg, Germany

## Abstract

**Background:**

Transposable elements are abundant in eukaryotic genomes and it is believed that they have a significant impact on the evolution of gene and chromosome structure. While there are several completed eukaryotic genome projects, there are only few high quality genome wide annotations of transposable elements. Therefore, there is a considerable demand for computational identification of transposable elements. LTR retrotransposons, an important subclass of transposable elements, are well suited for computational identification, as they contain long terminal repeats (LTRs).

**Results:**

We have developed a software tool *LTRharvest *for the *de novo *detection of full length LTR retrotransposons in large sequence sets. *LTRharvest *efficiently delivers high quality annotations based on known LTR transposon features like length, distance, and sequence motifs. A quality validation of *LTRharvest *against a gold standard annotation for *Saccharomyces cerevisae *and *Drosophila melanogaster *shows a sensitivity of up to 90% and 97% and specificity of 100% and 72%, respectively. This is comparable or slightly better than annotations for previous software tools. The main advantage of *LTRharvest *over previous tools is (a) its ability to efficiently handle large datasets from finished or unfinished genome projects, (b) its flexibility in incorporating known sequence features into the prediction, and (c) its availability as an open source software.

**Conclusion:**

*LTRharvest *is an efficient software tool delivering high quality annotation of LTR retrotransposons. It can, for example, process the largest human chromosome in approx. 8 minutes on a Linux PC with 4 GB of memory. Its flexibility and small space and run-time requirements makes *LTRharvest *a very competitive candidate for future LTR retrotransposon annotation projects. Moreover, the structured design and implementation and the availability as open source provides an excellent base for incorporating novel concepts to further improve prediction of LTR retrotransposons.

## Background

For decades it has been known that parts of eukaryote genomes are repetitive. The major fraction of repeats are transposable elements, which are spread throughout the genomes in an interspersed fashion and make up approx. 50% of the human genome [1] or even higher percentages in plant species. Transposable elements are classified in three groups according to their mode of mobilisation (transposition): LTR retrotransposons (retrovirus like elements), non-LTR retrotransposons, and DNA transposons. They are well described by their sequence features as analysed with molecular genetics methods and by sequence comparison. However, the molecular mechanisms of transposition are in most cases not fully understood and the elucidation of their functions is still a matter of discussion. In addition, there are more questions than answers regarding the origin of transposable elements and their role in evolution, especially their contribution to modification of genomes. The genome wide identification of transposable elements is not only an essential step in the annotation of genomes but also offers an opportunity to obtain in depth knowledge on features of transposable element families.

Repeat detection is a well studied problem in bioinformatics. It is mostly based on sequence analysis. A multitude of computational tools have been developed for an automated annotation of repeat families in sequenced genomes. Probably the best known program is *RepeatMasker *[2]. It screens DNA query sequences for interspersed repeats and low complexity DNA sequences. Precompiled sequence libraries and special scoring matrices are used to detect similar copies in the query sequence. Therefore, *RepeatMasker *is the first choice for repeat annotation in genomes, in which repeat families have already been well characterized, although annotating vertebrate genomes takes days of calculation time on a single computer.

Transposable element families are reported to be lineage-specific, e.g. half of all human repeats arose after the divergence of mouse and human and most repeats in the mouse genome are not found in the human genome [3]. *RepeatMasker*'s repeat libraries contain lots of repeat families from model organisms, while repeat libraries for non-model organisms exist with only limited curation [4]. In addition, transposable elements within a family might be highly divergent depending on the time of activity of the source repeat and in these cases identification by sequence comparison methods is not always successful. This holds especially when the search is performed over species borders. Therefore, automated *de novo *methods for repeat detection are desirable. Once for conducting a fast repeat detection, and additionally to speed up the cumbersome process of generating repeat libraries.

In most cases *de novo *methods for finding repeats start with a self-comparison to detect sequence similarities, followed by clustering methods to group related sequences into families. A number of widely used programs are already available for this task, e.g. *REPuter *[5] (or the improved and more general software tool *Vmatch *[6]), *RECON *[7], RAP [8] and PILER [9].

Besides their repetitive nature, most classes of transposable elements are characterized by more specific constraints. These can be, for example, distance and length constraints for repetitive sequences within the repeat element or some motif, that is typical for this repeat element. Thus, repeats identified by general repeat detection tools additionally have to be screened in order to find candidates satisfying criteria of their specific class of transposable element. To achieve high quality predictions it is necessary to build software for the individual classes of transposable elements.

LTR retrotransposons make up a large fraction of the interspersed repeats and are well classified by several structural attributes [10]. The long terminal repeats (LTRs) are the hallmark of canonical LTR retrotransposons and make them an ideal target for *de novo *prediction. Full length or nearly full length LTR retrotransposons bear the following features that might be suitable for *de novo *prediction: LTRs appear in a certain size range and distance between each other. In addition, LTRs are flanked by a short target site duplication (TSD). In some species, e.g. in yeast, the LTRs contain a conserved dinucleotide motif at their 5' and 3' end. The internal region contains genes important for retrotransposition and some conserved sequence motifs. However, depending on the age of the element, the LTR sequences, the open reading frames, and motifs are degenerated through mutations.

There are already some software programs specifically designed for the *de novo *LTR retrotransposon detection problem. *LTR_STRUC *[11] is the best known of these programs. *LTR_STRUC *has been applied to the genomes of the fruitfly *Drosophila melanogaster *[12], *Oryza sativa *(rice) [13], *Mus musculus *(mouse) [14] and recently *Pan troglodytes *(chimpanzee) [15]. The program *LTR_par *[16] follows a similar approach but uses a faster algorithm than *LTR_STRUC*. The *de novo *prediction of LTRs is also the first step in the recently developed software [17] (abbreviated LTR_Rho in this publication), and by the program LTR_FINDER [18]. Both programs consider further features of LTR retrotransposons in post processing steps to enhance the quality of the predictions.

The aim of this work is the development of a software tool, that efficiently works on large genomes and in addition is flexible in parameterization in order to be used for various species. Our program called *LTRharvest *implements the same LTR model as *LTR_par*, but uses a different composition of algorithms and features:

1) It allows for fast computation of large data sets, e.g. vertebrate genomes that are in the order of 2 – 3 gigabases sequences length.

2) Flexible parameter settings allows the user to incorporate biological features like LTR length and distance, TSD length and motifs.

3) *LTRharvest *accepts sequences in multiple FASTA format and is therefore able to work on whole genome shotgun (WGS) sequencing data, which usually come as multiple unordered contigs.

4) *LTRharvest *is open source software that can easily be modified and extended to satisfy further needs.

Here we present a comprehensive introduction to the software tool *LTRharvest *and show several benchmarks. *LTRharvest *was validated on yeast and fruitfly genome annotations with very good results in both sensitivity and specificity of the predictions. We also compare the quality and performance of *LTRharvest *to existing software tools *LTR_STRUC *[11], *LTR_par *[16], LTR_Rho [17] and LTR_FINDER [18]. This benchmark demonstrates that *LTRharvest *is considerably faster and memory efficient with a prediction quality as good as or even better then the aforementioned software tools.

## Implementation

### Features of LTR retrotransposons used for *de novo *prediction

An autonomous LTR retrotransposon (for a review see [10]), that bears all features essential for retrotransposition is composed of two nearly identical LTR sequences which are flanked by TSDs of usually 4 – 6 bp. In some species, small palindromic motifs at the 5' and 3' end of the LTRs are observed. The internal region is composed of several open reading frames, for example the *pol *gene encodes for protease, reverse transcriptase (RT) and integrase (IN) enzymatic functions and the *gag *gene encodes structural proteins for the virus-like particle. In rare cases, an *env-like *gene, that is essential for the retroviruses life cycle, is present in LTR retrotransposons although not essential for retrotransposition. Finally, conserved sequence motifs, e.g. the so-called primer binding site (PBS) that acts as starting point for the reverse transcription and a purine rich sequence called the poly purine tract (PPT) is observed at the 3' end of the internal region. All these features may be used for *de novo *predictions. However, in a particular genome some or most of the LTR retrotransposons may not have these features due to mutations they acquired during evolution. Point mutations lead to full length or nearly full length elements with degenerate LTR sequences as well as disruption of the internal open reading frames. Large deletions or insertions of other sequences, mainly from other transposable elements, are often observed and lead to truncated or nested LTR retrotransposons, respectively. Such LTR retrotransposon elements lack the canonical features to a certain extent and may be missed with *de novo *prediction software. Another common degenerated product are the so called solo LTRs, which consist of only one LTR due to exchange between the two LTRs flanking an element.

**Figure 1:**
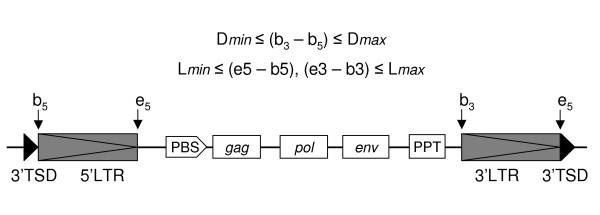
**Structure of a typical LTR retrotransposon/retrovirus (adapted from [16])**. LTR = long terminal repeat, TSD = target site duplication, PBS = primer binding site, PPT = poly purine tract, gag, pol, env = open reading frames for LTR retrotransposon genes.

For computational detection, structural attributes of LTR retrotransposons are modeled in *LTRharvest *following the model of [11] and [16] (see also Figure 1):

- Length constraint: The number of nucleotides between the start and end positions of an LTR is bounded within a range [L_*min*_, L_*max*_].

- Distance constraint: The number of nucleotides separating the start positions of the two LTR instances is bounded within the ranges [D_*min*_, D_*max*_].

- Similarity constraint: 5' and 3' LTR sequences normally show a high sequence identity. For newly inserted LTR retrotransposons the LTR sequences are identical due to the specific reverse transcription mechanism. Over evolutionary time, the LTRs can undergo mutations and therefore may differ more or less in their sequences.

- Target site duplications (TSDs): The regions 4–6 bp immediately upstream and downstream of the 5' and 3' LTR respectively normally show a high sequence identity, but may have aquired mutational variations over evolutionary time.

- LTR motif: Often LTR sequences start and end with a short conserved motif consisting of two nucleotides that form a palindromic sequence when joined, i. e. tg...ca.

In summary, *LTRharvest *is designed to detect LTR retrotransposon candidates that contain at least two LTRs. Solo LTRs, truncated elements that lack one LTR, or elements with large insertions do not fulfill the model underlying *LTRharvest*. However, such copies can be detected in a postprocessing step e.g. by homology searches of the computed LTR retrotransposons in the genome under investigation.

### Algorithms and workflow underlying *LTRharvest*

**Figure 2:**
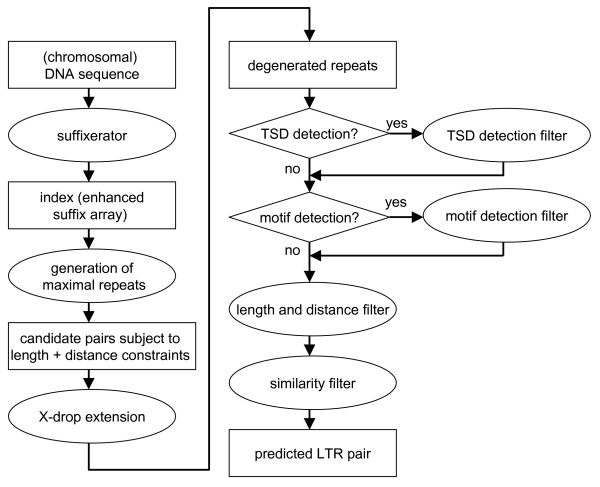
**Flowchart of the LTR retrotransposon prediction process in the program *LTRharvest***. The optional clustering process used in some benchmark tests is not shown in this flowchart.

The first step in the work flow of *LTRharvest *(Figure 2) is the construction of an enhanced suffix array for the genome or chromosome under consideration. As input, *LTRharvest *can also handle sequences in multiple FASTA format, such as those from WGS sequencing data. *LTRharvest *considers each sequence of a (multiple) FASTA file independently. That is, it does not search across boundaries of a sequence. Ambiguous characters (N, S, Y, W, R, K, V, B, D, H, M) are treated such that they do not match anywhere (not even themselves).

We use the program suffixerator which is part of the GenomeTools [19]. This enhanced suffix array requires 5 n bytes of memory, where n is the length of the input sequence. In contrast to other software for LTR retrotransposon prediction the enhanced suffix array is stored on file. Thus the most time consuming preprocessing step is separated from the LTR detection phase. This saves considerable time when processing the same genome with different parameter settings.

The software tool *LTRharvest *maps the enhanced suffix array into the main memory and begins searching for maximal exact repeats, using the linear time algorithm of [20]. The minimum length of the maximal exact repeats can be specified by the user. Maximal exact repeats satisfying user defined length and distance constraints are further processed to determine degenerated repeats. This is done by extending the seeds to the left and to the right by the greedy dynamic programming algorithm of [21]. This algorithm, called X-drop extension algorithm, runs considerably faster than traditional dynamic programming algorithms, while still delivering optimal alignments. The user defined drop-off parameter X allows to prune the search space: The smaller X, the smaller the search space and the more similar the aligned regions are. For maximum flexibility, we implemented the X-drop algorithm with user defined scores for deletions, insertions, and replacements.

The degenerated repeats, called candidate pairs, are then subject to the detection of LTR retrotransposon specific features, namely TSDs and palindromic LTR motifs. Both features are optional. TSDs of a user specified minimum and maximum length are searched in the left and right vicinity of the 5' and 3' instance of a candidate pair, respectively. To efficiently search for TSDs, *LTRharvest *extracts the corresponding two sequence sections from the genome. We consider the smaller of these sequence sections a reference and the larger a query. For the reference, *LTRharvest *constructs the suffix array in main memory and runs the standard search algorithm of [22] to find all exact matches of the query and the reference with length greater than or equal to the minimum TSD length. The TSD-search (if switched on) rejects all candidate pairs lacking the TSD. All other candidate pairs are processed further.

The palindromic LTR motif consists of two pairs of two nucleotides and an allowed number of mismatches between these. If the optional TSD filter is switched off, all candidate pairs are searched for this motif using a linear scan of the sequences at the outer boundaries of the candidate pair. In case the TSD filter is switched on, the candidate pairs with TSDs are searched by a simple character comparison at the TSD boundaries. A candidate pair without the palindromic LTR motif is rejected. All other candidate pairs are further processed.

Finally, LTRharvest checks for each remaining candidate pair whether the user specified LTR distance and length constraints are satisfied. Additionally, it is checked whether the LTR sequences including the TSDs and motifs (corresponding to the candidate pair) have a user defined minimal sequence identity. This is calculated by the greedy alignment algorithm of [23].

If all filters are passed, then the candidate pair is a predicted LTR retrotransposon. *LTRharvest *reports its start and end position as as well as some additional information of the prediction (e.g. the positions of the TSDs, the motif, the similarity, the sequence) in tabular format, in GFF format (version 3) [24] and/or FASTA format.

## Results and Discussion

### Rationale for usage of *LTRharvest*

As outlined before, *LTRharvest *is designed for fast detection of LTR retrotransposons in larger genomes. The computationally expensive step of building the enhanced suffix array is carried out before the search of LTR pairs and reporting of LTR retrotransposon candidates. For example, building the enhanced suffix array for each of the 24 human chromosomes (total size of ~3000 Mb) takes approximately one hour. However, searching for LTR retrotransposon candidates takes only minutes. So, the user can easily and within reasonable time run several predictions for different parameter sets.

Flexible parameters are of great advantage in case the user has previous knowledge about the common features of LTR retrotransposons in the species under investigation. This knowledge may come from the annotation results of a closely related species or from several LTR retrotransposons already sequenced in the species under investigation. In *LTRharvest *parameters can be adjusted to meet the species specific attributes of LTR retrotransposons.

The high quality annotations of transposable elements in *Saccharomyces cerevisiae *[25] and of *Drosophila melanogaster *[26] illustrate such species specific constraints. For example, in yeast 50 of 331 LTR retrotransposons are full length or nearly full length elements [25] and are suitable for *de novo *prediction. These yeast LTR retrotransposons were identified by sequence comparison to 5 LTR retrotransposon families and share > 70% sequence identity to the respective query sequence. Average size of the LTRs and of full length copies is quite similar between the LTR retrotransposon families. Nearly all of the 50 full length or nearly full length elements contain a TSD. In contrast, 682 LTR elements were annotated in *Drosophila melanogaster *and grouped into 49 families [26]. 304 of the 682 LTR elements were classified as 'full length' elements, while all elements less than 97% of the length of the canonical member of their family were classified as partial elements. Pairwise comparison of members within these families showed > 92% sequence identity [26]. In another study the comparison of LTR retrotransposon elements to canonical sequences showed higher divergences of up to 17% [27]. The size of the canonical elements of the 49 families varies between approx. 2483 and approx. 9092 bp [26]. TSD are observed within all LTR retrotransposon families, but sequence motifs at the 5' and 3' end of the LTRs are present in some but not all LTR retrotransposon families [28]. Therefore, the adjustment of parameter settings is important for *de novo *prediction and *LTRharvest *allows for flexible parameter choices set by the user. Of course, all filters can be switched off if desired.

Currently there are hundreds of genome sequencing projects for eukaryote species [29]. So the number of large sequence sets with unknown species specific features of LTR retrotransposons is increasing. *LTRharvest *is designed to be used on such genome data sets. It can handle multiple FASTA files from whole genome shotgun (WGS) sequence projects. In a typical trial on a genome, where limited or no biological features of LTR retrotransposons are known, flexible filter settings are of advantage in order to get an optimal result. The rationale of parameter optimization can be outlined as follows: The length and distance constraints regarding the LTRs should be set first. A major impact on the prediction result is obtained by setting proper parameters for seed length and X-drop extension of initial hits and similarity constraints. The user can either search in a strict manner to predict evolutionary young elements, where LTRs are nearly identical or relax the filters so that degenerated LTRs are also reported. The filters for TSD detection and LTR motifs should only be varied when special features of these parameters are known in advance.

When applying *LTRharvest *(or other *de novo *prediction software) to genomes with limited previous knowledge, a postprocessing is highly recommended to get rid of parts of the false positives. This can be carried out by various methods:

- Sequence clustering of the reported predictions, e.g. by running *Vmatch*, will bin most true positives in clusters while most false positives appear as singlets. The probability for a hit being a true positive prediction is increased if this sequence is located in a cluster. The division of sequences into clusters gives additional information about evolutionary relations of LTR retrotransposon families and their specific features or can serve as a start point for phylogenetic studies.

- Sequence based search for LTR specific protein domains (as done in LTR_Rho) or for sequence motifs (as done in LTR_FINDER).

- Sequence comparison of the reported hits to Repbase [2,4].

Like other *de novo *prediction software, *LTRharvest *predicts only full length or near full length elements showing canonical features like LTRs, TSDs and distance constraints. Once identified, the predicted LTR elements can be used in sequence similarity searches for the detection of further LTR retrotransposons including old, highly divergent, partial and nested LTR sequences as well as solo LTRs.

### Outline of the benchmark tests

For the evaluation of *LTRharvest *we performed two independent benchmarks. As benchmark sets we selected annotations of LTR retrotransposons from *Saccharomyces cerevisiae *[25] and *Drosophila melanogaster *[26] for the following reasons:

- The main body of the reference data was annotated by sequence comparison and not by other *de novo *prediction programs, that might have biased the reference set for 'easy to predict cases'.

- As the *de novo *predictions delivered by *LTRharvest *strongly depend on the detection of the 5' and 3' LTRs, we determine the specificity and the sensitivity of the result by comparison to individual LTR retrotransposons and not by comparison to consensus sequences of LTR retrotransposon families.

- As outlined above, *LTRharvest *cannot predict partial elements that lack parts of the LTRs or solo LTRs. 'Full length' LTR retrotransposon elements were extracted from the reference datasets according to the definitions given in the respective annotations [25,26].

- For the *D. melanogaster *we decided to work on the well documented data set of release 3 [26] although release 4 and 5 are already available and additional LTR retrotransposons were described by [30]. However, most of the additional elements are small fragments or are located in nested regions and therefore would not have been a target for *LTRharvest*.

The comprehensive survey of retrotransposons of *S. cerevisiae *has 50 known full length LTR retrotransposons [25] and is available from the *Voytas Lab Homepage *[31]. Unfortunately, there is no exact description of which version of the yeast genome was used by Kim et al. [25], but the annotation has probably been conducted on sequences released before Oct. 1st, 1997. Archived genome sequences were obtained from the ftp repository of the *Saccharomyces *Genome Database (SGD) [32] and can also be found at [33].

The Release 3 genomic sequences of *D. melanogaster *were obtained from the Berkeley *Drosophila *Genome Project (BDGP) ftp server [34]. This release is the first high-quality complete euchromatic sequence of the *D. melanogaster *genome and was used in the comprehensive genome-wide survey of transposable elements [26]. The *D. melanogaster *LTR retrotransposon annotation from Kaminker et al. [26] lists 49 LTR families with 682 LTR elements. Assuming that canonical elements represent full-length active copies, Kaminker et al. [26] defines any element less than 97% of the length of the canonical member of their family as partial LTR retrotransposons. According to this definition, 55% (i.e. 378) of the annotated LTR elements are classified as partial, 58 of these are solo LTRs and a large number of LTR elements only comprise one LTR sequence plus parts of the internal region. Of course, these elements cannot be found by *de novo *prediction software based on the prediction of repeats with length and distance constraints. Therefore, 304 LTR retrotransposons classified as 'full length' [26] served as dataset for the benchmark. These were extracted according to the 'full length' definition of Kaminker et al. [26]. In addition we used the complete dataset of 682 LTR retrotransposons marked as 'all' in our tests (data given the Additional file 1, Table C).

The data sets provided by Kaminker et al. [35] and the annotations provided by Flybase [36] were different with respect to the coordinates given for 70 out of 682 reported LTR retrotransposon insertions. The coordinates differ in most cases only by a few bases in the start or end position. As the specificity and the sensitivity of the predictions were calculated by comparison to individual LTR retrotransposon insertions, we defined an *LTRharvest *hit as true positive, if the 5' and 3' positions of the prediction and annotation match within a difference of ± 20 nucleotides. A half true positive (hTP) is a hit with one end matching within a difference ± 20 nucleotides and the other end not satisfying the definition of a true positive (TP). This strategy covered all annotation differences except for four cases: two are small partial insertions of < 400 bp and two nested hits < 900 bp and < 350 bp. The ± 20 nucleotides difference was also applied to the yeast data, as the annotation could not exactly be matched to the genome sequences.

In a first test *LTRharvest *predictions were compared to the data sets of annotated LTR retrotransposons in order to assign true positives (TP), false positives (FP) and false negatives (FN). The sensitivity is calculated by dividing the number of TP by the sum of all TPs and FNs (sensitivity = TP/(TP + FN)). For calculating of the specificity, all TPs were divided by the sum of all TPs and FPs (specificity = TP/(TP + FP)). In a second benchmark, we compared *LTRharvest *to four other LTR prediction programs in terms of quality and runtime. Again tests were carried out with datasets of *S. cerevisiae *and *D. melanogaster*. Finally, we checked if the different software tools are able to process the largest human chromosome. Prediction coordinates and coordinates from the reference annotation were automatically compared by Python scripts [33].

Parameter choices used for *LTRharvest are *shown in Table 1 and described here for the *D. melanogaster *data set. The parameters for the minimum and maximum LTR length and distance were optimized by plotting the corresponding values of LTR sequences from TPs and FNs after some test runs (data not shown). To optimize the initial seed length, FNs from test runs were used in an iterative process. Reasonable values for a maximal seed length should be within the range [20,100] bp, since the length of LTR sequences is expected to within the range [100,1000] bp. If the rate of mutation in the host genome is expected to be high, a short seed length, for instance 30 bp, should be combined with a large X-drop extension parameter, for instance 7, 8 or 9. If the rate of mutation in the host genome is expected to be low, a long seed length, for instance 80 bp, should be combined with a low X-drop extension parameter, for instance 3, 4 or 5. Table 1 shows reasonable values for the match, mismatch, insertion and deletion scores of the X-drop extension algorithm determined in various experiments. It has been known from literature that, in most cases, the distance between the two LTRs of a LTR retrotransposon is bounded by a minimum distance of 1000 bp and maximum distance of 15000 bp. For finding evolutionary young elements, the similarity threshold should be set at a high level, for instance 75%. The filters for TSD detection and LTR motifs should only be varied when special features are known in advance. The motif filter was not applied as there are several family specific 5' and 3' end LTRs motifs known in fruit flies.

**Table 1:** Parameter sets for *LTRharvest *used for predictions in genomes of *S. cerevisiae *and *D. melanogaster*

**Parameter name**	**Default**	**Value S. cer.**	**Value D. mel.**	**Comment**
seed	30	100	76	exact match length requirement for 5'-3' LTR pair
minlenltr	100	100	116	length constraint for 5'-3' LTR pair, in bp
maxlenltr	1000	1000	800	length constraint for 5'-3' LTR pair, in bp
mindistltr	1000	1500	2280	distance constraint for 5'-3' LTR pair, in bp
maxdistltr	15000	15000	8773	distance constraint for 5'-3' LTR pair, in bp
similar	85%	80.0%	91.0%	similarity threshold of a 5'-3' LTR pair, in bp
xdrop	5	5	7	Xdrop score for extending seed
mat	2	2	2	match score
mis	-2	-2	-2	mismatch score
ins	-3	-3	-3	insertion score
del	-3	-3	-3	deletion score
mintsd	4	5	4	minimal length of a target site duplication (TSD), in bp
maxtsd	20	20	20	maximal length of a target site duplication (TSD), in bp
motif	-	tg...ca	-	required motif
motifmis	0	0	0	maximum number of mismatches in motif
vic	60	60	60	number of nucleotide positions (vicinity) to the left and to the right, respectively, for searching TSD and motif around boundaries
overlaps	best	best	best	strategy for handling predicted LTR elements which overlap

column 'value S. cer.' = setting for *S. cerevisiae*

column 'value D. mel.' = setting for *D. melanogaster*

For a detailed description of parameters, see the manual of *LTRharvest *[33].

### Comparison of *LTRharvest *predictions to reference datasets

All predictions of *LTRharvest *on the data sets of *S. cerevisiae *were checked by comparing the genomic positions of the individual LTR retrotransposons and, in addition, by sequence comparison of the TSD and LTR motifs to ensure that these hits are true positives. All parameters settings (see Table 1) were determined by manually optimizing the parameters as outlined in the section above. *LTRharvest *predicted a total of 45 full-length LTR elements (90% sensitivity and 100% specificity). Five LTR retroelements were not accurately detected, as they did not pass at least one filter: 4 FNs do not have TSDs, 3 FNs do not possess the LTR end motif tg..ca and in one case a large insertion/deletion in the LTRs was observed. *LTRharvest *did not report any FPs.

For the *D. melanogaster *genome *LTRharvest *predicted a total of 505 elements (Table 2). Of these 279 predictions were TPs with half of these matching precisely at the 5' and 3' boundary coordinates of the corresponding LTR retrotransposon element. 20 predictions are hTPs for which the median difference in distance was 43 positions for the boundary coordinate not occurring in the 20 bp range. Five FNs were analysed in more detail. Four FNs are not flanked by TSDs with one of these having additional highly divergent LTR sequences and, therefore, did not fulfil the seed length conditions. Finally one FN (FBti0020325) mistakenly had been rejected in exchange for a false prediction with a higher similarity value. It should be noted, that 4 of the 5 FNs are annotated as being a 'member of nest' [26], where exact predictions are difficult due to the nested location of several transposable elements. However, *LTRharvest *located further 12 retrotransposons that are 'member of nest' as TP and two elements as hTPs.

**Table 2:** Quality validation of running *LTRharvest *on the *D. melanogaster *genome sequences (release 3)

**Chr**	**Predictions**	**References**	**TP**	**hTP**	**FP**	**FN**
2L	91	66	64	1	26	1
2R	89	54	49	3	37	2
3L	96	59	51	7	38	1
3R	96	67	60	6	30	1
4	11	4	4	0	7	0
X	122	54	51	3	68	0
**Total**	**505**	**304**	**279**	**20**	**206**	**5**

The used options are listed in Table 1 in column 'value D. mel.'. Column 'Predictions' gives the number of predicted and column 'References' the number of annotated 'full length' LTR retrotransposons for each chromosome. A prediction is classified as true positive (TP), if the maximum allowed distance between 5' and 3' coordinates of the prediction and the reference is at most 20 nucleotides. If only one of the two predicted boundary coordinates lies within the allowed distance of 20 nucleotides, the prediction is categorised as a half true positive (hTP). All other predictions are labelled false positive (FP). LTR retrotransposons that are missing in the prediction are labelled false negative (FN).

Since only the dataset of known full-length elements was used for calculation of sensitivity and specificity, there could be several partial LTR elements among the large amount of 206 FPs. Indeed, 94 FPs turned into 69 TP and 25 hTPs when compared to all 682 LTR retrotransposons. Thus 94 FPs are partial LTR retrotransposons. A BLAST search [37] of the remaining 112 FPs against a database of all transposable elements of *D. melanogaster *[35] showed that most of these FPs contain in part LTR retrotransposon sequences, but are not predicted with the exact position or show in part sequence identity to non-LTR transposons or DNA transposons. In addition some FPs contain tandem repeats.

When comparing to the 'full length' annotation, *LTRharvest *achieved a sensitivity of 91.7% counting all TPs and 98.4% counting TPs and hTPs, respectively. The specificity is 55.2% counting TPs only, and 59.2% counting hTPs and TPs. It should be noted, that a considerable portion of FPs are partial LTR elements.

When applying *LTRharvest *on genomes without annotation data regarding transposable elements, a separation of predictions in TPs and FPs is, of course, not possible. In this case, an automated separation of the predicted sequences by a classification into sequence families is desirable. A single linkage cluster analysis on the set of all 505 predictions was conducted using *Vmatch*. The clustering process resulted in 36 clusters with 421 out of 505 (83%) elements in clusters and 84 out of 505 (17%) elements as singlets. Such singlets seem unlikely to contain LTR retrotransposons and would be excluded from further studies. Elements in the 36 clusters were compared to the reference LTR retrotransposon families reported by Kaminker et al. [26], which consist of 41 families with at least one full-length member and 8 families with only partial elements (see Table 3). 34 out of the 41 full-length families (and 1 of the 8 partial families) were identified by the clustering of *LTRharvest *predictions, where three clusters consist of more than one reference LTR retrotransposon family. This result demonstrates that *LTRharvest *in combination with a clustering of the predicted sequences is suitable for *de novo *detection of LTR retrotransposons. Seven clusters consist completely of FPs (see Table 3). A BLAST sequence comparison to the *D. melanogaster *genome sequences showed that LTRharvest_Dmel18, -22, and -23 showed a hit on single loci within the genome organised in tandem repeats. LTRharvest_Dmel27 and -30 showed several hits on a single loci with several repeats to regions containing a LTR and a non-LTR retrotransposon, respectively. LTRharvest_Dmel31 matched to a genomic region containing several nested transposable elements whereas LTRharvest_Dmel35 showed several hits to the LTR retrotransposon of the family GATE. For the predictions delivered by *LTRharvest *and followed by clustering (and removal of singlets), the sensitivity is 97.4% and the specificity is 70.3% counting TPs and hTPs together.

**Table 3:** List of clusters of *LTRharvest *predictions compared to the *Drosophila melanogaster *annotation

**LTR retrotransposon predictions from *LTRharvest *and clustering**		***Drosophila melanogaster *annotation of LTR retrotransposon families from [26]**		
**cluster**	**# sequences in cluster**	**name of family**	**full-length sequences**	**all sequences**

LTRharvest_Dmel0	92	Roo	58	146
LTRharvest_Dmel1	18	opus	16	24
LTRharvest_Dmel2	15	mdg1	13	25
LTRharvest_Dmel3	2	McClintock	2	2
LTRharvest_Dmel4	22	blood	22	22
LTRharvest_Dmel5	26	412	24	31
LTRharvest_Dmel6	50	297,17.6	25	69
LTRharvest_Dmel7	12	Stalker, Stalker2, Stalker4	9	27
LTRharvest_Dmel8	3	rover	3	6
LTRharvest_Dmel9	15	micropia, DM88, invader1	3	63
LTRharvest_Dmel10	3	invader3	3	16
LTRharvest_Dmel11	19	Tirant	15	20
LTRharvest_Dmel12	28	copia	26	30
LTRharvest_Dmel13	9	diver	9	9
LTRharvest_Dmel14	6	Quasimodo	5	14
LTRharvest_Dmel15	5	Transpac	5	5
LTRharvest_Dmel16	3	Idefix	2	7
LTRharvest_Dmel17	12	Burdock	7	13
LTRharvest_Dmel18	3			
LTRharvest_Dmel19	16	blastopia	13	17
LTRharvest_Dmel20	6	springer	5	11
LTRharvest_Dmel21	12	HMS-Beagle	9	13
LTRharvest_Dmel22	3			
LTRharvest_Dmel23	5			
LTRharvest_Dmel24	3	GATE	0	20
LTRharvest_Dmel25	8	mdg3	8	16
LTRharvest_Dmel26	4	invader2	3	10
LTRharvest_Dmel27	2			
LTRharvest_Dmel28	4	3S18	4	6
LTRharvest_Dmel29	2	gypsy5	1	2
LTRharvest_Dmel30	3			
LTRharvest_Dmel31	2			
LTRharvest_Dmel32	2	gypsy, gtwin	3	8
LTRharvest_Dmel33	2	invader4	2	9
LTRharvest_Dmel34	2	Tabor	2	3
LTRharvest_Dmel35	2			

For clustering the program *Vmatch *was used with the following options: seedlength for minimal length of exact repeats = 50, minimal length of matches = 2500, Xdrop = 9 and matching conditions (dbcluster) that cover at least 80% of the smaller sequence and 28% of the larger sequence.

Of 49 annotated families [26], this Table lists 34 out of 41 families containing at least one full length member and one family (GATE) out of 8 families that are entirely composed of partial elements.

### Comparison of *LTRharvest *to other LTR prediction software tools

We compared *LTRharvest *with the *de novo *LTR retrotransposon prediction tools *LTR_STRUC *[11], *LTR_par *[16], LTR_Rho [17], and LTR_FINDER [18]. Instead of *LTR_par*, we obtained a Linux-binary *LTR_seq *from the authors. *LTR_seq *is the sequential version of *LTR_par *and delivers the same results (S. Aluru, personal communication). As recommended by the authors, we split the input sequence for *LTR_seq *into small overlapping parts to improve the run-time. For each tool, the set of genome sequences and annotations from *S. cerevisiae *and *D. melanogaster *was used as described in the previous section. All *LTR_STRUC *runs were conducted on a Windows-XP PC with an Intel Core2 processor (CPU 2.00 GHz, 4096 KB cache size, 2 GB memory). The other test runs were performed on a Linux system with an Intel Xeon processor (CPU 2.66 GHz, 512 KB cache size, 1 GB memory).

Each test-run has a unique 'run-number', that is used in Tables 4 and 5 as well as in the Tables B and C of the Additional file 1. For completeness, a documentation of the parameter settings used in each run and exact numbers of TPs, hTPs, FPs and FNs are given in the Additional file 1. LTR_FINDER uses a filter based on a set of species specific precompiled tRNA sequences by default. The program was run with this filter (no. 4-1) and without this filter (no. 4-2, data only shown in the Additional file 1). LTR_Rho additionally uses an HMM search for retrotransposon specific open reading frames to enhance the prediction quality. We used *LTRharvest *without clustering (no. 5-1) and with clustering as a post processing step (no. 5-2). Prediction coordinates and coordinates from the reference annotation were automatically compared by Python scripts [33]. The benchmark result is presented in terms of program run-time, sensitivity, and specificity calculated as described above. For *D. melanogaster*, the sensitivity and specificity was evaluated for the 'full length' and the 'all' dataset. It should be noted, that some LTR retrotransposons are not included in the 'full length' data set. Hence for the 'full length' dataset none of the programs achieves 100% specificity. However, the 'full length' data set allows for a fair comparison of the sensitivity, as solo LTRs and highly degenerated LTR retrotransposons not suitable for *de novo *prediction are excluded.

**Table 4:** Quality validation of programs for LTR retrotransposon prediction on the genome of *S. cerevisiae*

**Program used**	***LTR_STRUC***	***LTR_seq***	**LTR_Rho**	**LTR_FINDER**	***LTR harvest***	***LTR_seq***	**LTR_Rho**	**LTR_FINDER**	***LTR harvest***
Run-number	1	2	3	4-1	5	6	7	8	9
Parameter set	default*	default	default	default	default	see Tab.1	see Tab.1	see Tab.1	see Tab.1
Index files contruction [s]	-	-	-	-	8	-	-	-	8
Run-time [s]	~600	413	190	19	3	126	168	19	2
Annotations	50	50	47	50	50	50	46	50	50
Predictions	39	50	46	56	68	38	38	43	45
Sensitivity	76%	80.0%	89.4%	100%	98.0%	74.0%	69.6%	84.0%	90.0%
Specificity	97.4%	100.0%	91.3%	89.3%	72.1%	97.4%	84.2%	97.7%	100%
Comment			program error chr03,06				program error chr03, 06,09		

* = parameters are not adjustable.

Details on parameter settings and exact numbers of predictions are shown in Table B of the Additional file 1. Sensitivity and specificity values were calculated as outlined in the *S. cerevisiae *benchmark counting all TPs and hTPs as true positives. In run-no. 2 and no. 7, the program LTR_Rho reported an error for some chromosomal sequences. Thus the number of annotations and predictions was adjusted to the incomplete data set.

**Table 5:** Quality validation of programs for LTR retrotransposon prediction on the genome of *D. melanogaster*

**Program used**	***LTR_STRUC***	***LTR_seq***	**LTR_Rho**	**LTR_FINDER**	***LTR harvest***	***LTR harvest***	***LTR_seq***	**LTR_Rho**	**LTR_FINDER**	***LTR harvest***
Run-number	1	2	3	4-1	5-1	5-2	6	7	8	9
Parameter set	default*	default	default	default	default	default + clustering	see Tab. 1	see Tab.1	see Tab. 1	see Tab.1 + clustering
Index files contruction [s]	-	-	-	-	138	138	-	-	-	138
Run-time [s]	4380	24120	2286	1209	25	198**	1380	1709	320	170**
**Annotations 'full length'**	304	304	304	304	304	304	304	304	304	304
Predictions	310	188	417	395	723	490	160	398	204	411
Sensitivity	37.5%	36.8%	94.7%	74.3%	94.7%	97.4%	35.2%	96.1%	52.0%	97.7%
Specificity	36.8%	59.6%	69.1%	57.2%	40.4%	60.4%	66.9%	73.4%	77.5%	72.3%
**Annotations 'all'**	682	682	682	682	682	682	682	682	682	682
Predictions	310	188	417	395	723	490	160	398	204	411
Sensitivity	20.1%	22.0%	54.0%	45.9%	58.9%	57.9%	19.8%	53.5%	28.7%	56.3%
Specificity	44.2%	79.8%	88.2%	90.3%	55.6%	80.6%	84.4%	91.7%	96.1%	93.4%

* = parameters are not adjustable.

** = run-time *LTRharvest *+ clustering with *Vmatch*.

Details on parameter settings and exact numbers of predictions are shown in Table C of the Additional file 1. Sensitivity and specificity values were calculated against the *D. melanogaster *annotation counting all TPs and hTPs as true positives.

All tools were first run with their default parameter settings (no. 1 – 5 in Tables 4 and 5). Because these settings varied considerably (see Additional file Table B and C), we then used parameter settings as similar as possible to the settings specified in Table 1 (no. 6 – 9). As different parameter settings, especially the seed length, influence the run-times, these are compared for run no. 6 – 9. Since *LTR_STRUC *parameters are not adjustable, the performance of this tool with the parameter settings according to Table 1 could not be evaluated. In four final runs we used the default settings of the various programs for *LTRharvest *(no. 10 – 13 in the Additional file).

Using the default settings of each program for predicting yeast LTR retrotransposons, LTR_FINDER and *LTRharvest *show the best sensitivity with 100% and 98% respectively. The specificity of *LTRharvest *is low compared to the other programs. However, using of the parameter settings according to Table 1, the specificity of *LTRharvest *improves. Run-time comparison of the programs (no 1, 6 – 9 in Table 4) show, that *LTRharvest *is the fastest program (10 seconds) followed by LTR_FINDER (19 seconds), *LTR_seq *(126 seconds), LTR_Rho (168 seconds) and *LTR_STRUC *(600 seconds). This may partly be due to the fact, that *LTRharvest *and LTR_FINDER are completely implemented as a single C and C++ binary, respectively, while LTR_Rho is a Perl script gluing together several other programs. *LTR_STRUC *may be so slow because it uses several brute force algorithms.

The evaluation of the programs for the *Drosophila *genome gives a different picture. When comparing *LTRharvest *with LTR_STRUC and *LTR_seq *(all three programs use the same basic model), *LTRharvest *is clearly the best in terms of prediction quality and run-time. The sensitivity of LTR_Rho and *LTRharvest *is in the same range, while the high specificity of LTR_Rho is only reached by *LTRharvest *with the specific parameter settings for *Drosophila *(see Table 1) and a clustering step. However, it should be noted, that LTR_Rho predicted 84 and 94 hTPs, respectively, (no. 3 and no. 7, Additional file 1, Table C) in contrast to *LTRharvest *with only 14 and 20 hTPs, respectively (no. 5-2 and no. 9, Additional file 1, Table C). LTR_Rho's inaccurate detection of LTR element boundaries may be explained by a missing TSD search procedure, which seems important for an exact boundary detection.

Parameter settings from the different programs of this benchmark were used for *LTRharvest *(no. 10 – 13, Additional data file, Table C). While these settings did not much affect the sensitivity, the specificity of the predictions varied between 30.0% and 61.0% for the 'full length' annotation. Clustering as a post processing step considerably enhances the specificity of *LTRharvest *predictions (see no. 5-1 vs. no. 5-2 vs. no. 9 in Table 5). For the 'all' annotation, the specificity of *LTRharvest *with clustering (no. 9) is 93.4%.

The fastest program for *D. melanogaster *is *LTRharvest *and clustering (308 seconds, no. 9, Table 5) followed by the LTR_FINDER (320 seconds, no. 8 in Table 5). Of the test runs producing results with sufficient quality (sensitivities above 70% and specificity above 50%), the second best program is LTR_Rho with 1709 seconds run-time (no. 7, Table 5). In summary, using optimised parameter settings and clustering, *LTRharvest *gave the best results in this benchmark test.

### Test runs on a complete chromosomal human genome sequence

All software tools were tested to see if they are capable of processing a sequence of size of a vertebrate chromosome. As test sequence, the complete sequence of human chromosome 2 from Build 36.2 with a length 242,951,149 bp was used. All programs were run with their default settings. Test runs were performed on a PC with 2 GB (for the Windows XP operating system) and 4 GB (for the SUSE Linux 10.2 operating system) main memory. Only *LTR STRUC, LTR_seq *and *LTRharvest *were able to handle the chromosome sequence and terminated successfully after ~480, ~240, and ~8 minutes, respectively. In case of *LTRharvest*, the construction of the enhanced suffix tree took ~5 minutes and required a maximum of 1218.67 MB of main memory. The LTR prediction took 67 seconds using default settings and 154 seconds when including the motif search (see Table 1, column S. cer.). LTR_Rho's binary for the detection of maximal repeats terminated with an error message ('ERROR: No MEM') most likely indicating insufficient memory. LTR_FINDER terminated with a 'segmentation fault'.

## Conclusion

The goal of this work was to develop a run-time efficient and space efficient LTR retrotransposon detection software tool delivering high quality predictions. The basic model of the LTR retrotransposon structure was taken from McCarthy and McDonald [11] and from Kalyanaraman and Aluru [16]. Based on this model efficient and flexible filter algorithms, different from those used in [11] and [16], were implemented in the software program *LTRharvest*. The results from the validation of *LTRharvest *are encouraging. In all test-runs, *LTRharvest *showed a sensitivity of at least 90% on the *S. cerevisiae *genome and more than 96% on the *D. melanogaster *genome for the detection of full-length LTR retrotransposons. Moreover, *LTRharvest *achieves a high level of specificity (> 93% and > 72% for the 'all' and the 'full length' annotations, respectively) if clustering is carried out as a post processing step. Together, the *LTRharvest *prediction and the clustering process represent a strong method for obtaining a high quality *de novo *annotation of full length or near full length LTR retrotransposons. As holds true for other *de novo *prediction tools, short partial LTR retrotransposon copies, solo LTRs and some nested elements cannot be predicted by *LTRharvest*. However, these copies can be identified by a sequence similarity search using *de novo *identified species specific LTR retrotransposons. *LTRharvest *showed fast run-times and low memory consumption enabling *de novo *prediction for large data sets like vertebrate chromosomes. As the time consuming step of building the enhanced suffix array has to be carried out only once for a dataset, iterative predictions using different parameter settings e.g. for improving sensitivity or specificity are fast and easy to perform.

The source code, a test dataset and the manual of *LTRharvest *can be found at the homepage of the Center for Bioinformatics Hamburg [33]. We provide precompiled binaries for Linux and Mac OS X (Intel). The manual includes a detailed description of the filters and their parameters. All parameters can be specified by command line options.

Future improvements of *LTRharvest *will focus on the implementation of further filters checking for the presence of LTR retrotransposon specific open reading frames, primer binding sites (PBS) or poly purine tracts (PPT).

## Availability and requirements

**Project name**: *LTRharvest*

**Project home page**: 

**Operating system(s)**: POSIX compliant UNIX systems, for example Linux, Mac OS X, Solaris and OpenBSD

**Programming language**: C

**Other requirements**: *GNU *C compiler and *GNU *make for compiling the source code; bash and python for running the example scripts

**License**: BSD-like open source licences, see 

**Any restrictions to use by non-academics**: none

## Authors' contributions

UW and SK conceived the concept of the software and the benchmarks. SK suggested the algorithmic structure. DE developed the software and performed the benchmarks. All authors wrote and approved the manuscript.

## Supplementary Material

Additional file 1 **Full description of parameters used and results of the comparison of *de novo *LTR retrotransposon prediction programs**. Additional file 1 includes Table A – list of abbreviations, Table B – Quality validation on the *S. cerevisiae *genome (complete data) and Table C – Quality validation on the *D. melanogaster *genome (complete data). Click here for file
