# Supplementary material for: LTRharvest, an efficient and flexible software for de novo detection of LTR retrotransposons
Source: BMC Bioinformatics. 2008 Jan 14;9:18. doi: 10.1186/1471-2105-9-18 (PMC2253517; doi:10.1186/1471-2105-9-18)
Supplement: Additional file 1 — Full description of parameters used and results of the comparison of de novo LTR retrotransposon prediction programs. Additional file 1 includes Table A – list of abbreviations, Table B – Quality validation on the S. cerevisiae genome (complete data) and Table C – Quality validation on the D. melanogaster genome (complete data). [file 1471-2105-9-18-S1.pdf]

Table A: Abbreviations used in Table B and C

Abbreviation	Explanation
parameter set	It names whose program’s default settings were used. (Parameters were adjusted as far as possible.)
Lex	Minimal length for maximal repeats.
Lmin, Lmax	Minimal and maximal length constraints for LTRs.
Dmin, Dmax	Minimal and maximal distance constraints for LTRs.
LTR similarity	Similarity threshold for LTRs.
LTR motif search	Search for LTR motifs.
LTR motif necessary	Predictions must have a specified motif.
TSDs search	Search for TSDs.
TSDs necessary	Predictions must have TSDs.
seed extension	Information about the seed extension parameters.
clustering	Clustering process of predicted sequences using the *Vmatch* single linkage clustering program:<br><br>parameter name, value, comment<br>dbcluster, 95 ((Dmin / Dmax) x 100 +1) , match covers at least 95% of the smaller and (formula value)% of the larger sequence<br>d, , compute direct matches<br>p, , compute palindromic matches<br>seedlength, 50, minimal length of exact repeats<br>l, Dmin + Lmin, minimal length of matches<br>exdrop, 9, Xdrop score used in seed extension
tRNA	Use of precompiled tRNA sequences for detection of PBS.
PPT search	Search for PPT (poly purine tract) using dynamic programming techniques.
RT search	Search for RT (reverse transcriptase) domains using dynamic programming techniques.
IN, RnaseH search	Search for IN (integrase), RnaseH domains using dynamic programming techniques.
generic ORF search	Search for an open reading frame.
HMMsearch	Search for a particular ORF (open reading frame) using RT-, RNaseH-, IN-and Protease-HMMs.
annotations	Number of annotated elements in the corresponding benchmark set.
Index files construction	Construction time in seconds for all index files, i.e., the enhanced suffix array that is stored on files for the input sequences, only used by *LTRharvest*
run-time	Real run-times were determined by the unix program time, except for LTR STRUC where run-times were determined from the log-data files.
predictions	Number of predicitions made by the program.
allowed ± difference	Allowed ± difference from exact position for categorising a prediction asa true positive.
TP	Number of true positives.
hTP	Half true positives with only one boundary coordinate predicted correctly.
Total of TP	Sum of TP and hTP.
FP	Number of false positives.
FN	Number of false negatives.
Sensitivity	Sensitivity of prediction, calculated by TP/(TP + FN)
Specificity	Specificity of prediction, calculated by TP/(TP + FP)

Table B: Quality validation on the entire *S. cerevisiae* genome (complete data set).

Parameters marked by the asterisk '*' were fixed (not adjustable) in the corresponding tool.

Run-time marked with '**' is for *LTRharvest* + clustering with V*match* with the options given in Table A.

**Program used**	LTR_STRUC	*LTR_seq*	LTR_Rho	LTR_FINDER	LTR_FINDER	*LTRharvest*	*LTR_seq*	LTR_Rho	LTR_FINDER	*LTRharvest*	*LTRharvest*	*LTRharvest*	*LTRharvest*	*LTRharvest*
**Run-number (new)**	1	2	3	4-1	4-2	5	6	7	8	9	10	11	12	13
Parameter set	default*	default	default	default	default	default	see Tab.1	see Tab.1	see Tab.1	see Tab.1	*LTR_seq*	LTR_Rho	LTR_FINDER	LTR_FINDER
L*ex*	40*	30	40	20	20	30	100	100	100	100	30	40	20	20
L*min*	?	100	1	100	100	100	100	100	100	100	100	1	100	100
L*max*	?	2000	1000	3500	3500	1000	1000	1000	1000	1000	600	1000	1000	1000
D*min*	?	600	1100	1000	1000	1000	1500	1500	1500	1500	600	1100	1000	1000
D*max*	?	15000	16000	20000	20000	15000	15000	15000	15000	15000	15000	16000	20000	20000
LTR similarity	70*	85	80	70	70	85	80	80	80	80	85	80	70	70
LTR motif search	yes*	yes	no*	yes*	yes*	no	yes	no*	yes*	yes	yes	no	no	no
LTR motif necessary	no*	tg..ca	no*	no	no	no	tg..ca	no*	tg..ca	tg..ca	tg..ca	no	no	no
TSDs search	yes*	yes	no*	yes*	yes*	yes	yes	no*	yes*	yes	yes	yes	yes	yes
TSDs necessary	no*	yes	no*	no	no	yes	yes	no*	yes	yes	yes	yes	yes	yes
Seed extension	default*	default	default*	default	default	default	see Tab. 1	default*	default	see Tab.1	see Tab.1	see Tab.1	see Tab.1	see Tab.1
Clustering	no*	no*	no*	no*	no*	no*	no*	no*	no*	no	no*	yes	no	yes
tRNA	yes*	no*	no*	yes	no	no*	no*	no*	no	no*	no*	no*	no*	no*
PPT search	yes*	no*	no*	yes*	yes*	no*	no*	no*	yes*	no*	no*	no*	no*	no*
RT search	yes*	no*	no*	yes*	yes*	no*	no*	no*	yes*	no*	no*	no*	no*	no*
IN, RnaseH search	no*	no*	no*	no	no	no*	no*	no*	no	no*	no*	no*	no*	no*
Generic ORF search	yes*	no*	yes*	no*	no*	no*	no*	yes*	no*	no*	no*	no*	no*	no*
HMMsearch	no*	no*	yes*	no*	no*	no*	no*	yes*	no*	no*	no*	no*	no*	no*
**Results**														
Index files contruction [s]	-	-	-	-	-	8	-	-	-	8	8	8	8	8
Run-time [s]	~ 600	413	190	19	19	3	126	168	19	2	3	10**	3	13**
Allowed +-difference	20	20	20	20	20	20	20	20	20	20	20	20	20	20
Predictions	39	50	46	56	48	68	38	38	43	45	50	50	81	50
Annotations [full length]	50	50	47	50	50	50	50	46	50	50	50	50	50	50
TP	38	40	39	50	44	47	37	23	42	45	45	46	47	43
hTP	0	0	3	0	0	2	0	9	0	0	0	2	2	2
Total of TP + hTP	38	40	42	50	44	49	37	32	42	45	45	48	49	45
FP	1	0	4	6	4	19	1	6	1	0	5	2	32	5
FN	12	10	5	0	6	1	13	14	8	5	5	2	1	5
Sensitivity	76%	80.0%	89.4%	100%	88.0%	98.0%	74.0%	69.6%	84.0%	90.0%	90.0%	96.0%	98.0%	90.0%
Specificity	97.4%	100.0%	91.3%	89.3%	91.7%	72.1%	97.4%	84.2%	97.7%	100%	90.0%	96.0%	60.5%	90.0%
Comment			program error chr03,06					program error chr03,06,09						

Table C: Quality validation on the entire *D. melanogaster* genome (complete data set).

Parameters marked by the asterisk '*' were fixed (not adjustable) or not availiable in the corresponding tool.

Run-time marked with '**' is for *LTRharvest* + clustering with V*match* with the options given in Table A. Settings marked by ? are not given in the manual.

**Program used**	LTR_STRUC	*LTR_seq*	LTR_Rho	LTR_FINDER	LTR_FINDER	*LTRharvest*	*LTRharvest*	*LTR_seq*	LTR_Rho	LTR_FINDER	*LTRharvest*	*LTRharvest*	*LTRharvest*	*LTRharvest*	*LTRharvest*
**Run-number (new)**	1	2	3	4-1	4-2	5-1	5-2	6	7	8	9	10	11	12	13
Parameter set	default*	default	default	default	default	default	default	see Tab.1	see Tab.1	see Tab.1	see Tab.1	*LTR_seq*	LTR_Rho	LTR_FINDER	LTR_FINDER
L*ex*	40*	30	40	20	20	30	30	76	76	76	76	30	40	20	20
L*min*	?	100	1	100	100	100	100	116	116	116	116	100	1	100	100
L*max*	?	2000	1000	3500	3500	1000	1000	800	800	800	800	200	1000	1000	1000
D*min*	?	600	1100	1000	1000	1000	1000	2280	2280	2280	2280	600	1100	1000	1000
D*max*	?	15000	16000	20000	20000	15000	15000	8773	8773	8773	8773	15000	16000	20000	20000
LTR similarity	70*	85	80	70	70	85	85	91	91	91	91	85	80	70	70
LTR motif search	yes*	no	no*	yes*	yes*	no	no	no	no*	yes*	no	no	no	no	no
LTR motif nesessary	no*	no	no*	no	no	no	no	no	no*	no	no	no	no	no	no
TSDs search	yes*	yes	no*	yes*	yes*	yes	yes	yes	no*	yes*	yes	yes	yes	yes	yes
TSDs necessary	no*	yes	no*	no	no	yes	yes	yes	no*	no	yes	yes	yes	yes	yes
Seed extension	default*	default	default*	default	default	default	default	see Tab. 1	default*	default	see Tab.1	see Tab.1	see Tab.1	see Tab.1	see Tab.1
Clustering	no*	no*	no*	no*	no*	no*	yes	no*	no*	no*	yes	no*	yes	no	yes
tRNA	yes*	no*	no*	yes	no	no*	no*	no*	no*	no	no*	no*	no*	no*	no*
PPT search	yes*	no*	no*	yes*	yes*	no*	no*	no*	no*	yes*	no*	no*	no*	no*	no*
RT search	yes*	no*	no*	yes*	yes*	no*	no*	no*	no*	yes*	no*	no*	no*	no*	no*
IN, RnaseH search	no*	no*	no*	no	no	no*	no*	no*	no*	no	no*	no*	no*	no*	no*
Generic ORF search	yes*	no*	yes*	no*	no*	no*	no*	no*	yes*	no*	no*	no*	no*	no*	no*
HMMsearch	no*	no*	yes*	no*	no*	no*	no*	no*	yes*	no*	no*	no*	no*	no*	no*
**Results**															
Index files contruction [s]	-		-	-	-	138	138		-	-	138	138	138	138	138
Run-time [s]	4380	24120	2286	1209	1208	25	198**	1380	1709	320	170**	49	196**	45	298**
Allowed +-difference	20	20	20	20	20	20	20	20	20	20	20	20	20	20	20
Predictions	310	188	417	395	278	723	490	160	398	204	411	768	484	987	519
**Annotations [full-length]**	304	304	304	304	304	304	304	304	304	304	304	304	304	304	304
TP	112	109	204	214	183	283	282	105	198	152	277	279	275	275	273
hTP	2	3	84	12	7	15	14	2	94	6	20	13	20	21	21
Total of TP + hTP	114	112	288	226	190	298	296	107	292	158	297	292	295	296	294
FP	196	76	129	169	88	425	194	53	106	46	114	476	189	691	225
FN	190	192	16	78	114	6	8	197	12	146	7	12	9	8	10
Sensitivity	37.5%	36.8%	94.7%	74.3%	62.5%	98.0%	97.4%	35.2%	96.1%	52.0%	97.7%	96.1%	97.0%	97.4%	96.7%
Specificity	36.8%	59.6%	69.1%	57.2%	68.3%	41.2%	60.4%	66.9%	73.4%	77.5%	72.3%	38.0%	61.0%	30.0%	56.6%
**Annotations [all]**	682	682	682	682	682	682	682	682	682	682	682	682	682	682	682
TP	129	139	255	270	223	364	360	125	244	182	343	360	349	352	347
hTP	8	11	113	43	28	38	35	10	121	14	41	37	47	51	49
Total of TP + hTP	137	150	368	313	251	402	395	135	365	196	384	397	396	403	396
FP	173	38	49	82	27	321	95	25	33	8	27	371	88	584	123
FN	545	532	314	369	431	280	287	547	317	486	298	285	286	279	286
Sensitivity	20.1%	22.0%	54.0%	45.9%	36.8%	58.9%	57.9%	19.8%	53.5%	28.7%	56.3%	58.2%	58.1%	59.1%	58.1%
Specificity	44.2%	79.8%	88.2%	79.2%	90.3%	55.6%	80.6%	84.4%	91.7%	96.1%	93.4%	51.7%	81.8%	40.8%	76.3%
